# MDMA-assisted psychotherapy for treatment of anxiety and other psychological distress related to life-threatening illnesses: a randomized pilot study

**DOI:** 10.1038/s41598-020-75706-1

**Published:** 2020-11-24

**Authors:** Philip E. Wolfson, Julane Andries, Allison A. Feduccia, Lisa Jerome, Julie B. Wang, Emily Williams, Shannon C. Carlin, Evan Sola, Scott Hamilton, Berra Yazar-Klosinski, Amy Emerson, Michael C. Mithoefer, Rick Doblin

**Affiliations:** 1 Center for Transformational Psychotherapy, San Anselmo, CA USA; 2 MAPS Public Benefit Corporation, Santa Cruz, CA USA; 3 Department of Psychiatry, University of California, San Francisco, CA USA; 4 Private Practice, San Francisco, CA USA; 5 Stanford School of Medicine, Stanford Stroke Center, Palo Alto, CA USA; 6 Multidisciplinary Association for Psychedelic Studies, Santa Cruz, CA USA; 7 Medical University of South Carolina, Charleston, SC USA

**Keywords:** Drug discovery, Medical research

## Abstract

The success of modern medicine creates a growing population of those suffering from life-threatening illnesses (LTI) who often experience anxiety, depression, and existential distress. We present a novel approach; investigating MDMA-assisted psychotherapy for the treatment of anxiety in people with an LTI. Participants with anxiety from an LTI were randomized in a double-blind study to receive MDMA (125 mg, n = 13) or placebo (n = 5) in combination with two 8-h psychotherapy sessions. The primary outcome was change in State-Trait Anxiety Inventory (STAI) Trait scores from baseline to one month post the second experimental session. After unblinding, participants in the MDMA group had one open-label MDMA session and placebo participants crossed over to receive three open-label MDMA sessions. Additional follow-up assessments occurred six and twelve months after a participant’s last experimental session. At the primary endpoint, the MDMA group had a greater mean (SD) reduction in STAI-Trait scores, − 23.5 (13.2), indicating less anxiety, compared to placebo group, − 8.8 (14.7); results did not reach a significant group difference (*p* = .056). Hedges’ *g* between-group effect size was 1.03 (95% CI: − 5.25, 7.31). Overall, MDMA was well-tolerated in this sample. These preliminary findings can inform development of larger clinical trials to further examine MDMA-assisted psychotherapy as a novel approach to treat individuals with LTI-related anxiety.

**Trial Registration**: clinicaltrials.gov Identifier: NCT02427568, first registered April 28, 2015.

## Introduction

Individuals facing, or who have faced, a life-threatening illness (LTI), contend with more than just the physical symptoms of their condition. Anxiety, depression, anger, and despair often exacerbate the distress already caused by the illness itself, even after a remission or cure is achieved^1^. It is common for survivors to harbor fears of potential relapse, recurrence, and death^2^. The trauma of a devastating illness is often deep and difficult to integrate into moving on with one’s life^3,4^. Additionally, the impact of LTIs on family, health care providers, and community can be profound and affect recovery. A significant increase in caregiver distress is also prevalent^1,5^. There is a great need for new treatment options to address the psychological distress associated with LTIs. The social and personal burden of the immense numbers of people surviving LTIs necessitates our full attention and care.

Early investigations with psychedelic compounds such as lysergic acid diethylamide (LSD) suggested that psychoactive substances held promise in addressing distress, pain, and anxiety in people with LTIs^6,7^. Findings from studies reported from 2011 to 2016^8–12^ provide evidence for the use of psychedelics, specifically psilocybin and lysergic acid diethylamide (LSD), as an efficacious modality for the treatment of depression, anxiety, and psycho-existential distress among those with LTIs, including the terminally ill^13,14^. Randomized, placebo-controlled trials reported reduction in symptoms of anxiety and depression compared with controls, with some indication that symptom reduction might be linked to subjective drug effects, such as strength of a mystical experience^15^. Manualized 3,4-methylenedioxymethamphetamine (MDMA)-assisted psychotherapy shares a number of similarities with methods used in psychedelic-assisted psychotherapy.

MDMA is under investigation as an adjunct to psychotherapy for various anxiety-related conditions. Compelling results from six Phase 2 studies led the FDA to issue a Breakthrough Therapy designation for MDMA-assisted psychotherapy for treatment of posttraumatic stress disorder (PTSD) in 2017^16^. In the Phase 2 trials, participants who were given active-dose MDMA (75–125 mg) and psychotherapy experienced significantly greater reductions in PTSD symptoms when compared with participants given inactive placebo or low-dose MDMA (0–40 mg)^17–21^. MDMA-assisted psychotherapy also reduced symptoms of depression and improved sleep quality. A study of MDMA-assisted psychotherapy in autistic adults with social anxiety also found significantly greater improvement in Leibowitz Social Anxiety Scale (LSAS) Total scores in the MDMA group compared to the placebo group^22^.

MDMA stimulates release of monoamines (serotonin, dopamine, and norepinephrine), elevates levels of the neurohormone oxytocin, reduces amygdala and right insular activity in response to negative emotional stimuli, increases superior frontal cortex activity, and increases connectivity between the amygdala and hippocampus^23–26^. In such studies, functional magnetic resonance imaging (fMRI) technique, blood oxygen level dependent imaging, or BOLD-contrast imaging, was used to assess neuronal activity in these regions. The effects of MDMA may reduce anxiety in the face of emotionally challenging thoughts or memories and can increase self-compassion and enhance fear-extinction learning^27–30^. People with LTIs often experience anxiety and intrusive illness-related thoughts similar to symptoms of PTSD and may perceive or even develop PTSD from receiving an LTI diagnosis and/ or subsequent medical care. PTSD or PTSD-like symptoms are often reported after a cancer diagnosis, myocardial infarction, or stroke^31–33^; and several participants in previous study of MDMA-assisted psychotherapy have reported an LTI or a medical treatment to be comparable to an index trauma^20^. Considering the promising effects of MDMA-assisted psychotherapy in individuals with PTSD and social anxiety, a study was developed to assess MDMA-assisted psychotherapy in people with LTI-related anxiety.

The aim of this pilot study was to examine the safety and efficacy of MDMA-assisted psychotherapy, among patients with cancer or non-dementing neurological diseases, to alleviate anxiety and other psychiatric symptoms, including depression and poor sleep quality, related to an LTI. There preliminary results will serve to inform development of larger clinical trials.

## Results

**Figure 1:**
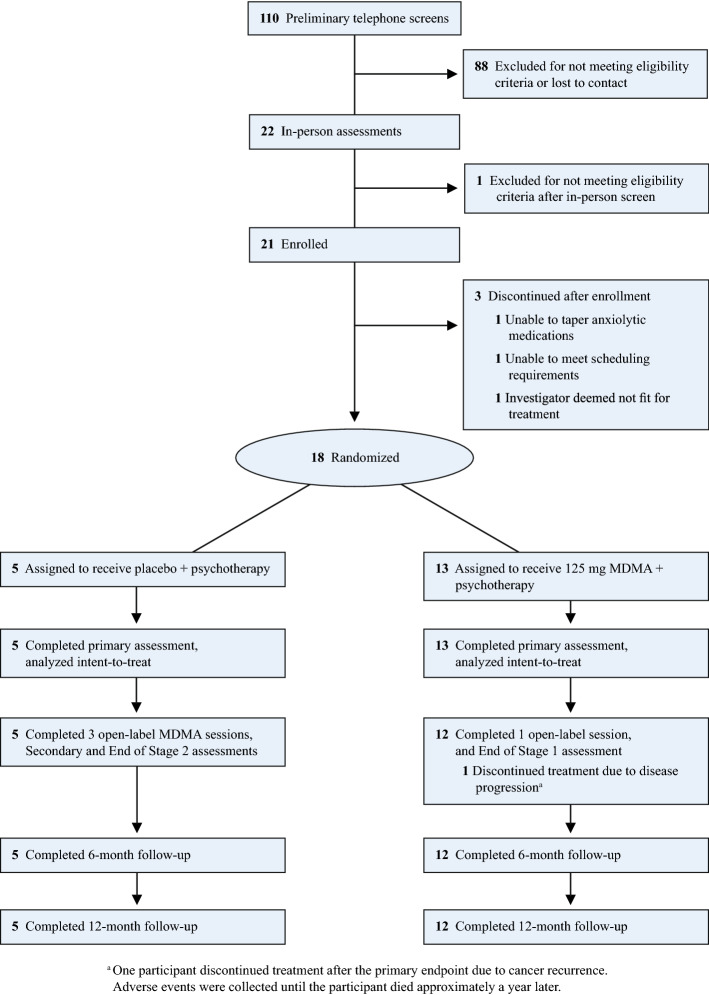
CONSORT diagram.

A total of 18 participants who met eligibility criteria were enrolled in the study between May 2015 to February 2017 and randomized to either receive MDMA (n = 13) or placebo (n = 5). Ninety-two of 110 participants who were initially screened failed to meet the inclusion criteria at telephone screening. The primary reasons for exclusion included not living in the study area and not being physically well enough, due to having a life-threatening illness, that prevented study participation. A few were lost to follow-up and three participants were excluded after enrollment and prior to randomization because they did not meet the study enrollment criteria (Fig. 1). Table 1 compares baseline characteristics between treatment groups. The overall sample had a mean (SD) age of 54.9 (7.9) years and was mostly female (77.8%) and White/Caucasian (83.3%). All participants had a prior diagnosis of an LTI. For the primary diagnosis for study inclusion, 94.4% had a diagnosis of neoplasms and one participant had a diagnosis categorized as a musculoskeletal and connective tissue disorder. Medical histories indicated that many of the participants were previously diagnosed with anxiety (83.3%), major depression (77.8%), PTSD (72.2%), or insomnia (61.1%). All participants were found to have moderate to severe anxiety at baseline, with a mean (SD) STAI-Trait score of 61.1 (7.0) and STAI-State score of 57.4 (10.9). Assessment of the Structured Clinical Interview for DSM-IV Axis Disorders—Patient Edition (SCID-I/P Version 2.0)^34^ during intake indicated that the baseline anxiety experienced by participants mostly stemmed from symptoms related to their LTIs. Seven of 18 participants (39.0%) reported taking an opioid medication during the course of the study. Six discontinued opiate medications at least three days prior to and two days after a blinded or unblinded MDMA session. One full-dose group participant reported taking a medication containing tramadol, an opiate with some serotonergic activity, during the course of the study but did not take the medication before, during, or within 24 h after an experimental session.

**Table 1:** Demographics and baseline characteristics.

	Placebo (n = 5)	MDMA (n = 13)	Total (n = 18)
Age, mean (SD), years	53.2 (10.5)	55.5 (7.0)	54.9 (7.9)
**Sex, no. (%)**			
Male	1 (20.0)	3 (23.1)	4 (22.2)
Female	4 (80.0)	10 (76.9)	14 (77.8)
**Race, no. (%)**			
White/Caucasian	3 (60.0)	12 (92.3)	15 (83.3)
Black/African-American	1 (20.0)	0	1 (5.6)
White/Native American	1 (20.0)	0	1 (5.6)
Other	0	1 (7.7)	1 (5.6)
BMI (kg/m^2^), mean (SD)	25.9 (3.2)	24.8 (4.0)	25.1 (3.8)
**Psychiatric medical history diagnosis**^**a**^,** no. (%)**			
Anxiety	3 (60.0)	12 (92.3)	15 (83.3)
Major depression	3 (60.0)	11 (84.6)	14 (77.8)
Posttraumatic stress disorder	2 (40.0)	11 (84.6)	13 (72.2)
Insomnia	2 (40.0)	9 (69.2)	11 (61.1)
Prior ecstasy use (yes), no. (%)	3 (60.0)	7 (53.9)	10 (55.6)
** Last use, no. (%)**			
2–5 years	1 (33.3)	1 (14.3)	2 (20.0)
6–10 years	1 (33.3)	1 (14.3)	2 (20.0)
> 10 years	1 (33.3)	5 (71.4)	6 (60.0)
**Pre-study psychotherapy type, no. (%)**			
EMDR	0	1 (7.7)	1 (5.6)
Group psychotherapy	2 (40.0)	1 (7.7)	3 (16.7)
Cognitive behavioral therapy	0	1 (7.7)	1 (5.6)
Psychodynamic	4 (80.0)	12 (92.3)	16 (88.9)
Interpersonal therapy	0	1 (7.7)	1 (5.6)
Holotropic breathwork	1 (20.0)	0	1 (5.6)
Other	3 (60.0)	6 (46.2)	9 (50.0)
None	0	0	0
**Lifetime C-SSRS**^**b**^, **no. (%)**			
Positive ideation	4 (80.0)	10 (76.9)	14 (77.8)
Serious ideation	1 (20.0)	0	1 (5.6)
Positive behavior	0	3 (23.1)	3 (16.7)
STAI-Trait^c^, mean (SD)	57.4 (5.2)	62.5 (7.3)	61.1 (7.0)
STAI-State^d^, mean (SD)	51.8 (5.3)	59.5 (11.9)	57.4 (10.9)

BMI, Body Mass Index; EMDR, Eye Movement Desensitization Reprocessing; C-SSRS, Columbia Suicide Severity Rating Scale.

^a^Medical history diagnosis: > 50% of participants indicated having these conditions.

^b^Lifetime accounts for all suicidal ideation and behavior prior to study according to participant recall and medical records. According to the C-SSRS scoring guide, scores of four or five on the suicidal ideation category are considered serious ideation and scores of one or greater are considered positive behavior or ideation.

^c^STAI-Trait: primary outcome measure of anxiety.

^d^STAI-State: secondary measure of anxiety.

**Figure 2:**
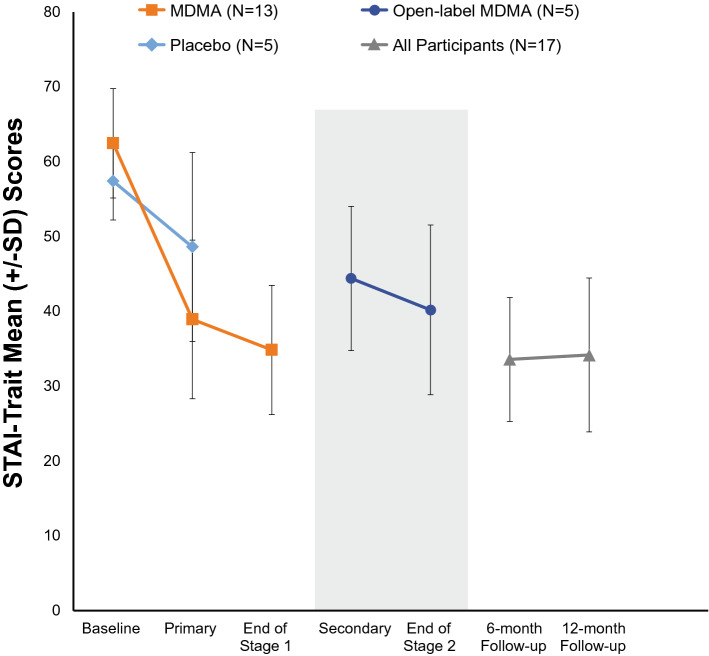
Mean (SD) STAI Trait scores for MDMA and Placebo groups at baseline and post treatment. Mean (SD) State Trait Anxiety Inventory scores across time at baseline, primary endpoint (one-month post second blinded experimental session), end of stage 1 (one month post third MDMA session, i.e. treatment exit for MDMA 125 mg group), secondary endpoint (one month post second open-label session), end of stage 2 (one month post third open-label session, i.e. treatment exit for control group), 6-month follow-up, and 12-month follow-up. The grey box represents the open-label crossover after placebo group was unblinded at the primary endpoint. Groups were collapsed for long-term follow-ups since all participants had received active doses of MDMA in either the blinded or open-label stage

The primary outcome was change in STAI-Trait anxiety scores^35^ from baseline to one-month post second blinded experimental session (Table 2, Fig. 2). The mean (SD) change in STAI-Trait anxiety score was greater for the MDMA group -23.5 (13.2) compared to the placebo group -8.8 (14.7), but these group differences were not statistically significant (*p* = 0.0558). The between-group Hedges’ *g* was 1.03 (95% CI: − 5.25, 7.31). In the placebo group, STAI-Trait anxiety change scores ranged from − 1 to − 35 with a median (IQR) of − 3 (1.0). One placebo participant had a STAI-Trait anxiety change score of − 35, which was well below the group median, and was therefore a potential outlier (data not shown). In comparison, in the MDMA group, STAI-Trait change scores ranged from − 43 to 1 with a median (IQR) of − 27 (13.0). If the one potential placebo outlier was removed, the STAI-Trait change scores between treatment groups in Stage 1 would have been statistically significant (*p* = 0.0066). Future studies with a larger sample size are needed to account for such outliers and elucidate these findings.

**Table 2:** Outcome measures^a^ at baseline and post two blinded experimental sessions.

	Placebo (n = 5)			MDMA (n = 13)			*p*-value
	Baseline	Post two experimental sessions	Change^b^	Baseline	Post two experimental sessions	Change^b^	
**Primary efficacy variable**							
STAI Trait, mean (SD)	57.4 (5.2)	48.6 (12.6)	− 8.8 (14.7)	62.5 (7.3)	38.9 (10.6)	− 23.5 (13.2)	0.06
**Secondary efficacy variables**							
STAI state, mean (SD)	51.8 (5.3)	45.8 (12.5)	− 6.0 (15.8)	59.5 (11.9)	37.5 (13.6)	− 22.1 (17.9)	0.10
BDI-II, mean (SD)	30.0 (11.4)	15.4 (9.9)	− 14.6 (8.6)	30.2 (11.0)	9.3 (10.4)	− 20.9 (13.8)	0.36
PSQI, mean (SD)	7.0 (6.6)	6.8 (5.7)	− 0.2 (1.3)	10.9 (3.5)	7.3 (4.5)	− 3.6 (5.4)	0.05
PTGI, mean (SD)	64.0 (19.1)	61.4 (24.9)	− 2.6 (6.1)	58.1 (19.9)	71.0 (18.8)	12.9 (23.2)	0.04
MADRS, mean (SD)	19.2 (9.3)	12.2 (5.3)	− 7.0 (7.2)	19.5 (7.1)	9.0 (9.0)	− 10.5 (8.2)	0.41
GAF, mean (SD)	69.8 (13.4)	72.8 (7.7)	3.0 (12.5)	68.5 (5.4)	75.1 (9.9)	6.6 (9.7)	0.52
SCS, mean (SD)	2.8 (0.8)	2.7 (0.9)	− 0.04 (0.5)	2.8 (0.6)	3.3 (0.6)	0.4 (0.7)	0.21
FFMQ, mean (SD)	3.3 (0.4)	3.3 (0.4)	0 (0.2)	3.3 (0.4)	3.7 (0.5)	0.4 (0.6)	0.04
DAP, mean (SD)							
Fear of death	5.1 (1.1)	4.5 (0.7)	− 0.6 (1.0)	3.8 (1.6)	3.7 (1.4)	− 0.1 (0.6)	0.25
Death avoidance	3.5 (1.9)	2.4 (0.9)	− 1.1 (1.8)	3.1 (1.7)	3.1 (1.6)	0 (0.8)	0.26
Neutral acceptance	5.4 (1.0)	5.6 (0.5)	0.2 (0.6)	5.8 (0.5)	5.9 (0.7)	0.1 (0.6)	0.88
Approach acceptance	3.2 (1.3)	3.0 (0.7)	− 0.1 (1.1)	3.2 (1.8)	3.5 (1.6)	0.3 (0.7)	0.32
Escape acceptance	3.4 (1.3)	3.4 (1.3)	0 (0.9)	3.5 (1.4)	3.9 (1.0)	0.4 (1.0)	0.85
FACIT, mean (SD)^c^							
Physical well-being	19.8 (6.7)	21.4 (3.0)	2.8 (5.0)	21.6 (4.2)	23.0 (4.3)	1.4 (4.4)	0.61
Social/family well-being	20.0 (9.7)	17.6 (6.3)	− 2.0 (2.9)	17.6 (2.9)	18.5 (3.8)	0.8 (3.4)	0.15
Emotional well-being	14.0 (5.9)	15.0 (3.9)	1.0 (2.2)	14.7 (3.0)	16.3 (6.7)	1.6 (7.1)	0.87
Functional well-being	19.5 (5.9)	18.8 (7.1)	1.0 (1.6)	14.5 (2.8)	19.3 (6.3)	4.8 (5.8)	0.22
Additional concerns	24.8 (14.4)	24.2 (10.3)	− 0.3 (5.0)	24.0 (9.2)	28.5 (14.1)	4.5 (11.9)	0.45

*STAI* State-Trait Anxiety Inventory, *BDI-II* beck depression inventory-II, *PSQI* Pittsburgh Sleep Quality Index, *PTGI* Post Traumatic Growth Inventory, *MADRS*, Montgomery-Asberg Depression Rating Scale, *GAF*, Global Assessment of Functioning, *SCS* Self-Compassion Scale, *FFMQ* Five-Facet Mindfulness Questionnaire, *DAP* Death Attitudes Profile, *FACIT* Functional Assessment of Chronic Illness Therapy Scale.

^a^All outcomes were based on an intent-to-treat set.

^b^Independent group *t*-test on change from baseline to post 2 experimental sessions.

^c^Missing FACIT data at baseline for placebo group (n = 4).

Secondary outcomes showed that the MDMA group significantly benefited vs. the placebo group for posttraumatic growth Post-traumatic growth Inventory^36^ (PTGI: Δ = 12.9, SD = 23.3 vs. Δ = − 2.6, SD = 6.1, *p* = 0.04, *g* = 0.50) and mindfulness Five Factor Mindfulness Questionnaire^37,38^ (FFMQ: Δ = 0.4, SD = 0.6 vs. Δ = 0, SD = 0.2, *p* = 0.04, *g* = 0.67). Results on the STAI-State anxiety, depression, sleep quality, and global functioning^39–44^ followed the same trajectory indicating greater improvement in the MDMA group vs. the control group but failed to reach statistically significant between-group differences (Table 2). After the open-label sessions, for both the MDMA and the placebo crossover groups, change scores improved across symptom domains (eTable 5). More specifically, among participants in the MDMA group, after their third and only unblinded MDMA session, mean (SD) STAI-Trait anxiety scores dropped nearly 4 points from their last (second) blinded MDMA session.

Table 3 presents change in outcome scores at baseline, treatment exit (after the last experimental session in stage 1 for the MDMA group and stage 2 for the placebo group), 6-month follow-up, and 12-month follow-up. After the crossover, by the end of the study, all participants had received similar treatment doses with three MDMA-assisted psychotherapy sessions. Therefore, groups were combined for one-way repeated measures ANOVA across time points, separately, for each outcome. Compared to baseline, nearly all outcomes improved from baseline to treatment exit and long-term follow-ups (Tukey’s pairwise comparison tests are presented in Table 3). In the overall ANOVAs, there were statistically significant reductions in STAI-Trait anxiety scores [F(3,48) = 51.39; *p* < 0.0001], STAI-State anxiety scores [F(3,48) = 34.19; *p* < 0.0001], BDI-II depressive symptom scores [F(3,48) = 18.74; *p* < 0.0001], and MADRS depression scores [F(3,48) = 47.30; *p* < 0.0001]. Participants reported statistically significant improvements in PSQI sleep quality [F(3,48) = 12.29; *p* < 0.0001], GAF global functioning [F(3,48) = 16.99; *p* < 0.0001], FACIT physical well-being [F(3,45) = 10.05; *p* < 0.0001], FACIT social and family well-being [F(3,45) = 3.58; *p* = 0.02], FACIT emotional well-being [F(3,45) = 22.71; *p* < 0.0001], FACIT functional well-being [F(3,45) = 20.27; *p* < 0.0001], FACIT additional concerns [F(3,45) = 26.93; *p* < 0.0001], DAP subscales fear of death [F(3,48) = 6.92; *p* = 0.0006], neutral acceptance [F(3,48) = 6.82; *p* = 0.0006], approach acceptance [F(3,48) = 4.03; *p* = 0.0123], and PTGI posttraumatic growth [F(3,48) = 23.45; *p* < 0.0001]. There were no significant changes for DAP subscales death avoidance [F(3,48) = 1.09; *p* = 0.36] and escape acceptance [F(3,48) = 1.28; *p* = 0.29]. Participants had statistically significant increases in SCS self-compassion [F(3,45) = 15.62; *p* < 0.0001] and FFMQ mindfulness [F(3,48) = 18.74; *p* < 0.0001].

**Table 3:** Outcome measures^a^ at baseline, treatment exit^b^, 6-month follow-up, and 12-month follow-up—within-subject.

	Placebo/MDMA^c^ (n = 5)	MDMA^d^ (n = 13)	Total^e^ (n = 17)	*p*-value^f^
**Primary efficacy variable**				
STAI trait score, mean (SD)				
Baseline	57.4 (5.2)	62.5 (7.3)	61.1 (7.0)	–
Treatment exit	40.2 (11.3)	34.8 (8.6)	36.4 (9.5)	< .0001
6-month follow-up	39.2 (11.1)	31.3 (5.9)	33.6 (8.3)	< .0001
12-month follow-up	36.8 (8.7)	33.1 (11.0)	34.2 (10.3)	< .0001
**Secondary efficacy variables**				
STAI state score, mean (SD)				
Baseline	51.8 (5.3)	59.5 (11.9)	57.4 (10.9)	–
Treatment exit	33.8 (10.2)	27.8 (6.5)	29.6 (8.0)	< .0001
6-month follow-up	33.0 (10.0)	29.2 (7.4)	30.3 (8.1)	< .0001
12-month follow-up	32.4 (8.6)	32.9 (12.4)	32.8 (11.1)	< .0001
BDI-II, mean (SD)				
Baseline	30.0 (11.4)	30.2 (11.0)	30.2 (10.8)	–
Treatment exit	3.8 (4.0)	2.7 (1.8)	3.0 (2.5)	< .0001
6-month follow-up	3.3 (2.5)	3.2 (3.3)	3.2 (3.1)	< .0001
12-month follow-up	6.8 (4.8)	3.3 (3.4)	4.3 (4.0)	< .0001
PSQI, mean (SD)				
Baseline	7.0 (6.6)	10.9 (3.5)	9.8 (4.7)	–
Treatment exit	6.2 (5.2)	5.7 (2.6)	5.8 (3.4)	0.0456
6-month follow-up	6.6 (5.7)	5.6 (3.9)	5.9 (4.3)	0.0412
12-month follow-up	5.2 (4.0)	6.3 (4.9)	5.9 (4.6)	0.0372
PTGI, mean (SD)				
Baseline	64.0 (19.1)	58.1 (19.9)	59.7 (19.3)	–
Treatment exit	82.0 (10.7)	83.8 (13.1)	83.3 (12.2)	< .0001
6-month follow-up	81.4 (10.3)	92.3 (9.8)	89.1 (10.9)	< .0001
12-month follow-up	81.6 (9.9)	89.3 (13.8)	87.0 (13.0)	< .0001
MADRS, mean (SD)				
Baseline	19.2 (9.3)	19.5 (7.1)	19.4 (7.5)	–
Treatment exit	4.2 (3.6)	4.1 (4.9)	4.1 (4.4)	< .0001
6-month follow-up	5.0 (3.1)	3.1 (2.2)	3.6 (2.5)	< .0001
12-month follow-up	4.6 (2.5)	3.0 (3.5)	3.5 (3.2)	< .0001
GAF, mean (SD)				
Baseline	69.8 (13.4)	68.5 (5.4)	68.8 (7.9)	–
Treatment exit	82.8 (6.5)	81.7 (6.0)	82.0 (6.0)	< .0001
6-month follow-up	80.0 (6.1)	84.1 (5.5)	82.9 (5.8)	< .0001
12-month follow-up	81.2 (7.0)	84.6 (7.2)	83.6 (7.1)	< .0001
SCS, mean (SD)^g^				
Baseline	2.8 (0.8)	2.8 (0.6)	2.8 (0.6)	–
Treatment exit	3.5 (0.7)	3.6 (0.7)	3.6 (0.7)	0.0065
6-month follow-up	3.3 (1.0)	3.8 (0.8)	3.7 (0.8)	0.0077
12-month follow-up	3.4 (0.9)	3.8 (0.8)	3.7 (0.8)	0.0254
FFMQ, mean (SD)				
Baseline	3.3 (0.4)	3.3 (0.4)	3.3 (0.4)	–
Treatment exit	3.7 (0.3)	3.9 (0.5)	3.9 (0.4)	< .0001
6-month follow-up	3.7 (0.4)	4.1 (0.5)	4.0 (0.5)	0.0002
12-month follow-up	3.8 (0.4)	4.1 (0.4)	4.0 (0.4)	0.0023
DAP, mean (SD)				
Fear of Death				
Baseline	5.1 (1.1)	3.8 (1.6)	4.1 (1.5)	–
Treatment exit	4.2 (1.3)	3.3 (1.6)	3.6 (1.5)	0.4540
6-month follow-up	4.0 (1.4)	3.1 (1.5)	3.4 (1.5)	0.4258
12-month follow-up	4.1 (1.6)	3.1 (1.5)	3.4 (1.6)	0.6867
Death avoidance				
Baseline	3.5 (1.9)	3.1 (1.7)	3.2 (1.7)	–
Treatment exit	2.3 (1.1)	2.8 (1.8)	2.6 (1.6)	0.7914
6-month follow-up	2.8 (1.6)	2.8 (1.8)	2.8 (1.7)	0.8806
12-month follow-up	2.6 (1.3)	2.7 (1.9)	2.7 (1.7)	0.7336
Neutral acceptance				
Baseline	5.4 (1.0)	5.8 (0.5)	5.7 (0.7)	–
Treatment exit	5.6 (0.6)	5.9 (0.6)	5.8 (1.0)	0.3139
6-month follow-up	5.9 (0.7)	6.3 (0.5)	6.2 (0.6)	0.1031
12-month follow-up	5.8 (0.6)	6.2 (0.4)	6.0 (0.5)	0.9588
Approach acceptance				
Baseline	3.2 (1.3)	3.2 (1.8)	3.2 (1.6)	–
Treatment exit	3.3 (0.7)	4.1 (1.4)	3.8 (1.3)	0.6365
6-month follow-up	3.4 (1.0)	4.0 (1.6)	3.8 (1.5)	0.5838
12-month follow-up	3.6 (0.8)	3.9 (1.6)	3.8 (1.4)	0.5687
Escape acceptance				
Baseline	3.4 (1.3)	3.5 (1.4)	3.5 (1.3)	–
Treatment Exit	3.4 (0.7)	4.2 (0.9)	4.0 (0.9)	0.8424
6-month follow-up	3.7 (0.8)	4.1 (1.5)	4.0 (1.3)	0.6101
12-month follow-up	3.8 (0.7)	3.9 (1.4)	3.8 (1.2)	0.6463
FACIT, mean (SD)^g^				
Physical well-being				
Baseline	19.8 (6.7)	21.6 (4.2)	21.2 (4.7)	–
Treatment exit	24.6 (3.4)	25.2 (3.5)	25.0 (3.4)	0.1204
6-month follow-up	23.0 (3.7)	25.3 (2.5)	24.6 (3.0)	0.0610
12-month follow-up	23.6 (4.0)	24.5 (4.9)	24.2 (4.5)	0.0320
Social and family well-being				
Baseline	20.0 (9.7)	17.6 (2.9)	18.2 (5.0)	–
Treatment exit	19.8 (6.1)	20.0 (3.8)	19.9 (4.4)	0.7207
6-month follow-up	17.6 (8.0)	21.2 (3.8)	20.1 (5.4)	0.6570
12-month follow-up	18.2 (7.1)	20.7 (3.6)	20.0 (4.8)	0.7207
Emotional well-being				
Baseline	14.0 (5.9)	14.7 (3.0)	14.5 (3.7)	–
Treatment exit	17.6 (3.4)	20.8 (2.0)	19.8 (2.8)	< .0001
6-month follow-up	19.0 (3.3)	20.6 (1.9)	20.1 (2.4)	< .0001
12-month follow-up	18.4 (2.7)	20.1 (3.1)	19.6 (3.0)	< .0001
Functional well-being				
Baseline	19.5 (5.9)	14.5 (2.8)	15.6 (4.1)	–
Treatment exit	21.8 (5.4)	21.8 (5.5)	21.8 (5.3)	< .0001
6-month follow-up	21.8 (5.0)	22.1 (3.0)	22.0 (3.6)	0.0005
12-month follow-up	21.4 (5.7)	23.5 (4.0)	22.9 (4.5)	0.0008
Additional concerns				
Baseline	24.8 (14.4)	24.0 (9.2)	24.2 (10.1)	–
Treatment exit	33.6 (8.9)	39.1 (7.6)	37.5 (8.1)	0.0002
6-month follow-up	30.8 (10.1)	40.1 (7.2)	37.4 (9.0)	0.0003
12-month follow-up	31.4 (8.9)	40.0 (6.3)	37.5 (8.0)	0.0002

*STAI* State-Trait Anxiety Inventory, *BDI-II* Beck Depression Inventory-II, *PSQI* Pittsburgh Sleep Quality Index, *PTGI* Post Traumatic Growth Inventory, *MADRS* Montgomery-Asberg Depression Rating Scale, *GAF* Global Assessment of Functioning, *SCS* Self-Compassion Scale, *FFMQ* Five-Facet Mindfulness Questionnaire, *DAP* Death Attitudes Profile, *FACIT* Functional Assessment of Chronic Illness Therapy Scale.

^a^All outcomes were based on an intent-to-treat set.

^b^Treatment Exit is defined as ‘after three MDMA sessions’ where MDMA group = End of Stage 1 and Placebo/MDMA group = End of Stage 2.

^c^Participants in the blinded placebo group crossed-over and received three open-label MDMA sessions.

^d^Participants in the blinded MDMA group had one MDMA session during open label.

^e^Baseline (n = 18), other endpoints (n = 17).

^f^Repeated measures ANOVA within subjects on time (*p* < 0.05); post-hoc contrasts between: Baseline to Treatment Exit; Baseline to 6-Month Follow-up; and Baseline to 12-Month Follow-up.

^g^Missing data: one participant missing data on SCS at 12-month follow-up (n = 16); one participant in placebo group missing data on FACIT (n = 4) at baseline.

MDMA was well-tolerated. The optional supplemental dose was taken in all but one session. The most commonly reported expected reactions during blinded MDMA administrations were thirst, jaw clenching/tight jaw, dry mouth, headache, and perspiration (Table 4). In the seven days following MDMA administration, the most frequently reported reactions were fatigue, needing more sleep, insomnia, and low mood, and these reactions decreased over the course of the week.

**Table 4:** Treatment-emergent adverse events and expected reactions during two MDMA Sessions and seven days following.

	Placebo (n = 5)	MDMA (n = 13)	Total (n = 18)
**Top reactions during experimental sessions, no. (%)**^**a**^			
Anxiety	0	3 (23.1)	3 (16.7)
Dry mouth	1 (20.0)	9 (69.2)	10 (55.6)
Headache	1 (20.0)	8 (61.5)	9 (50.0)
Insomnia	1 (20.0)	2 (15.4)	3 (16.7)
Jaw clenching, tight jaw	1 (20.0)	11 (84.6)	12 (66.7)
Lack of appetite	0	4 (30.8)	4 (22.2)
Nausea	1 (20.0)	3 (23.1)	4 (22.2)
Perspiration	0	9 (69.2)	9 (50.0)
Sensitivity to cold	1 (20.0)	2 (15.4)	3 (16.7)
Thirst	2 (40.0)	11 (84.6)	13 (72.2)
**Top reactions during 7 days of contact, no. (%)**^**a**^			
Anxiety	2 (40.0)	8 (61.5)	10 (55.6)
Drowsiness	1 (20.0)	6 (46.2)	7 (38.9)
Dry mouth	0	3 (23.1)	3 (16.7)
Fatigue	3 (60.0)	12 (92.3)	15 (83.3)
Increased irritability	1 (33.3)	2 (15.4)	3 (16.7)
Insomnia	2 (40.0)	9 (69.2)	11 (61.1)
Jaw clenching, tight jaw	1 (20.0)	8 (61.5)	9 (50.0)
Lack of appetite	0	4 (30.8)	4 (22.2)
Low mood	3 (60.0)	8 (61.5)	11 (61.1)
Nausea	1 (20.0)	6 (46.2)	7 (38.9)
Need more sleep	2 (40.0)	12 (92.3)	14 (77.8)
**Psychiatric TEAEs, no. (%)**^**b**^			
Anxiety	0	1 (7.7)	1 (5.6)
Depressed mood	0	1 (7.7)	1 (5.6)
Depression	0	1 (7.7)	1 (5.6)
Dissociation	0	1 (7.7)	1 (5.6)
Insomnia	1 (20.0)	2 (15.4)	3 (16.7)

*TEAEs* Treatment emergent adverse events.

^a^Frequency of subjects who reported an expected, spontaneously reported reaction collected during and seven days following blinded experimental sessions 1 and 2.

^b^Frequency of subjects who self-reported psychiatric adverse events after first drug administration until the day before experimental session 3.

During the blinded treatment period, Treatment Emergent Adverse Events (TEAEs) were infrequent and nearly equal between groups (Table 4). Most other TEAEs were likely associated with participants’ life-threatening illnesses (eTable 1). One participant discontinued treatment after the primary endpoint as a result of re-occurring cancer (unrelated to MDMA) and died approximately a year later. This participant experienced a series of five Serious Adverse Events (SAEs) that were associated with cancer recurrence and medical interventions, and included chordoma, spinal cord paralysis, meningitis, sepsis, and cerebrovascular accident. In addition, two other participants reported two SAEs related to cancer progression during the follow-up period.

Systolic blood pressure, diastolic blood pressure, heart rate, and body temperature (BT) generally increased more for the MDMA group, but only peak BT (*p* < 0.0001) reached significant differences between groups [mean (SD) BT: MDMA 37.3 °C (0.7) vs. Placebo 36.9 °C (0.3)] (eTable 2). Elevations in vital signs did not require any medical intervention and approached pre-drug values by session end. According to the C-SSRS, there were no reports of serious suicidal ideation or positive suicidal behavior during the study.

At the end of each blinded session, participants and co-therapy team members were asked to guess if MDMA or placebo was administered in the session. The investigators guessed correctly 32 of 36 (88.9%) sessions and incorrectly 4 of 36 (11.1%) sessions. Investigators guessed incorrectly for two participants, one assigned to placebo and one assigned to MDMA. Similarly, participants guessed correctly 31 of 36 (86.1%) sessions and incorrectly 5 of 36 (13.9%) sessions. There were three participants who guessed incorrectly. In 2 of 3 of these, the participant guessed MDMA when in fact they had received placebo.

## Discussion

The present study examined MDMA-assisted psychotherapy for individuals with moderate to severe anxiety associated with life-threatening illnesses. The primary analysis indicated participants who received MDMA-assisted psychotherapy had greater reductions in anxiety (STAI-Trait), compared to those in the placebo group, although group differences did not reach statistical significance (*p* = 0.056). In this study sample, lack of statistical significance was likely influenced by one potential outlier in the control group who had a particularly large reduction in their STAI-Trait score (change score of − 35) compared to the placebo group’s median (IQR) reduction of − 3 (1.0). Exclusion of this outlier rendered the group difference statistically significant (*p* = 0.0066). Additionally, 2 of 5 placebo participants believed they were in the MDMA group which might have produced a placebo effect. Therefore, a larger sample would be needed to adequately identify/mitigate the impact of outliers and other biases. At the primary endpoint, among the MDMA group, after two MDMA sessions, there were significant improvements in FFMQ mindfulness and PTGI total scores, an indicator of greater perceived benefits or positive effects after a difficult experience. Other symptom improvements in the MDMA group included depression, sleep quality, STAI-State anxiety, and global functioning. Results from the blinded portion of the study warrant larger clinical trials to examine MDMA-assisted psychotherapy as a novel approach to treat individuals who suffer from LTI-related anxiety. Data from these trials can also elucidate the relationship between outcome measures including identification of potential covariates that may mediate or moderate the primary outcome results.

After MDMA and Placebo/ MDMA group participants received three MDMA sessions, from baseline to treatment exit, the overall sample had improvements in anxiety, depression, sleep, global functioning, wellbeing (i.e., physical, social and family, emotional, functional), self-compassion, mindfulness, and attitudes regarding death. There were limitations in the long-term follow-up results, specifically, lack of a control group to eliminate the role of other factors in long-term benefits. However, at the 6- and 12-month follow-up visits, these outcomes were stable and above baseline levels, which suggests the potential for MDMA-assisted psychotherapy to produce long-term benefits of up to one or more years. Death Attitude Profile subscale scores improved for fear or death, neutral acceptance, and approach acceptance to suggest that some relief regarding participants’ attitudes about death could have reduced their LTI-related anxiety. These results were consistent with findings from a study on psilocybin-assisted psychotherapy, which reported people with LTIs had changes in death-related attitudes^9^. Participants’ attitudes towards death shifted after MDMA, as well as their daily coping mechanisms, as demonstrated by greater emotional and functional quality of life at the study endpoint. These preliminary findings suggest that MDMA-assisted psychotherapy might have the potential to provide long-term benefits for people who have or are overcoming a serious illness. Further research is also needed to examine possible mechanisms of MDMA-assisted psychotherapy including the role of potential mediators and moderators in reducing LTI-related anxiety.

There are several possible explanations for the effects of MDMA-assisted psychotherapy on anxiety and other symptoms. Previous studies have reported that PTSD can occur among people with chronic illnesses undergoing treatment, and that PTSD symptoms even persist long after remission^2,31,45,46^. A possible mechanism for MDMA reducing PTSD symptomology could be MDMA’s effect of decreasing amygdala activity, during presentation of negative stimuli, and increasing frontal lobe activity. In the current sample with an LTI, a large number of participants (72.2%) also had a PTSD diagnosis in their medical history. Although the index trauma for the PTSD diagnoses was not collected, it is likely that traumas and complex emotions were addressed through similar neural mechanisms and therapeutic processing. MDMA has previously been described as a “heart-opening” therapeutic substance^47,48^, which stimulates mindfulness, introspection, and empathy toward oneself and others. The effects of MDMA allow for empathic intervention of executive functions toward oneself and others^23,49,50^. Reduction in right insular activity may reduce anxiety through reducing attention and concern over the bodily experience of anxiety^25^. MDMA-induced changes in connectivity appear to be restricted to specific regions in the salience network, and not affecting connectivity globally. Other neurochemical and behavioral effects of MDMA include increased oxytocin^51^, elevated serotonergic activity^52^, increased self-compassion, and prosocial interactions with others^24,28^, which can enhance trust and rapport with therapists.

MDMA-assisted psychotherapy has demonstrated sustained reductions in PTSD symptoms in individuals who had failed to respond adequately to existing pharmacologic or psychotherapeutic treatments^17–22^. Compared to the placebo group in the blinded segment of the present study, the MDMA group trended toward reduced psychiatric symptoms, such as anxiety, depression and self-reported impaired sleep quality, associated with LTIs. While under the effects of MDMA, acute alterations in brain circuits important for in memory and emotional processing could have allowed participants to approach emotionally painful memories or thoughts with empathy and compassion rather than feeling overwhelmed by anxiety^28,49^. Prior studies in healthy adults have reported reduced negative ratings of person’s “worst” memories and increased vividness and intensity of emotionally positive memories after MDMA^28,49^. In the context of psychotherapy, this process may help people with LTIs by reducing fears of disease recurrence or death, and embracing compassion for self, others, and one’s situation. Individuals with LTIs who received three MDMA-assisted psychotherapy sessions showed long-term reduction in anxiety, assessed by MADRS at the 6- and 12-month follow-ups, and significant positive gains in posttraumatic growth or perceived benefits arising from an LTI, mindfulness (FFMQ), and social and family wellbeing (FACIT-S). The durability of improvement several months after MDMA-assisted psychotherapy sessions demonstrates benefits might extend beyond the acute treatment effects.

Consistent with previous reports from PTSD samples^17,18,20,21,53,54^, safety measures demonstrated that MDMA was well-tolerated by individuals with an LTI and no participants discontinued treatment due to adverse effects related to MDMA. After MDMA administration, MDMA group vital signs increased to expected levels, with only body temperature rising higher than the placebo group. The MDMA group also reported more adverse reactions during the experimental sessions, including jaw clenching/tight jaw, thirst, dry mouth, and perspiration. Reactions were short in duration and mostly subsided by the end of an experimental session, or during the week following. Psychiatric adverse events were infrequent, and MDMA was not associated with serious suicidal ideation or behavior. Overall, the safety profile for MDMA in this controlled clinical setting indicated that MDMA-assisted psychotherapy was a safe treatment in this relatively small sample where the benefits outweighed the cost of mild and short-term reactions. Future studies should continue to evaluate risks of adverse events in a larger sample of individuals with life-threatening illnesses.

Study limitations included the study design and small sample. This pilot study was exploratory and not powered to detect statistical significance. Additionally, the degree of group differences was impacted by an outlier in the placebo group who responded exceptionally well to psychotherapy alone compared to other participants in the placebo group during the blinded segment. This could have been influenced by a potential placebo effect, since 2 of 3 placebo participants believed they were assigned MDMA during the blinded sessions. In this relatively small study sample, this outlier might explain the lack of statistical significance in the between-group differences in primary outcome change scores, although a larger study would be needed to elucidate these findings. The study sample was mostly female and White/Caucasian, although males and persons of other ethnicities were also represented. After all participants received three MDMA sessions, results indicated significant improvements in outcomes at treatment exit, 6-month and 12-month endpoints. However, the interpretation of these results was limited due to lack of a control group after crossover.

## Conclusions

These findings provide preliminary evidence to support that MDMA-assisted psychotherapy may be a safe and feasible treatment for those with LTIs for anxiety reduction and relief of other psychiatric symptoms associated with their illness. Study results support the feasibility of MDMA-assisted psychotherapy as a novel approach for potential long-term treatment of LTI-related anxiety. These findings will inform development of future clinical trials with larger sample size and among more diverse populations.

## Methods

### Study design and participants

The present study was a Phase 2 clinical trial that tested the safety and efficacy of MDMA-assisted psychotherapy using a double-blinded, randomized, placebo-controlled design with an open-label crossover. The design consisted of a blinded segment that included two day-long experimental sessions (MDMA or placebo) scheduled two to four weeks apart, along with nine 60- to 90-min non-drug psychotherapy sessions; three preparing participants for the first experimental session and three for integration after each experimental session. Primary outcome measures were administered approximately one month after the second experimental session, and then the blind was broken. The design included a crossover segment where participants in the MDMA group had one additional open-label MDMA session and the placebo group participants had three open-label sessions with MDMA, with associated integrative sessions. The dose used in all MDMA sessions was 125 mg followed by an optional supplemental dose of 62.5 mg 90–150 min after the initial dose.

The study was conducted from April 2015 to May 2018 in an outpatient psychiatric clinic in San Anselmo, CA. The study protocol was approved by Western Copernicus Group Institutional Review Board and conducted in accordance with the ethical standards laid down in the 1964 Declaration of Helsinki. Participants were recruited through referrals from healthcare professionals, word-of-mouth, and internet advertisements. Eligible participants were men and women, aged 18 years or older, who were diagnosed with a life-threatening cancer or non-dementing neurological illness that was ongoing or in remission with risk of recurrence and had an estimated life expectancy of at least nine months. SCID-I/P was assessed at intake to evaluate whether a participant’s anxiety was primarily related to an LTI. Medical history information was collected through participant report and review of medical records. Participants had scores on the primary outcome measure, the State Trait Anxiety Inventory (STAI) Trait subscale of 45 (of 80), indicating moderate to severe anxiety.

Study exclusions were ongoing primary treatment for their illness, such as initial chemotherapy for cancer, major medical conditions contraindicated for MDMA administration, uncontrolled hypertension, history of significant cerebrovascular or cardiovascular disease, primary or metastatic tumors in the brain, renal disease, dementing neurological disease, diabetes type I or II, history of hyponatremia or hyperthermia, weight less than 48 kg, pregnancy (or lactation), diagnosis of psychotic disorders, bipolar disorder I, dissociative identity disorder, or eating disorder with active purging. Participants were excluded if they could not safely taper off psychiatric medications, which was required for study participation. Participants were permitted to take prescribed opiate medications. Enrollment was allowed for participants with substance use disorders, if in remission for at least 60 days prior to enrollment.

All participants provided written informed consent prior to enrollment in the study. Participants who gave written informed consent were screened for study eligibility and examined for non-psychiatric conditions by a physician.

### Randomization and masking

Participants were randomized to receive either inactive placebo (125 mg lactose) or 125 mg MDMA in an approximate 1:3 ratio using a web-based randomization system with unique container numbers. Randomization was maintained by individuals who monitored the randomization process without communicating with site staff, individuals monitoring the study, or data and statistical analysts. Participants’ group assignments were masked for participants, investigators, and an independent rater.

MDMA was manufactured by Dr. David Nichols (Purdue University, West Lafayette, IN, USA). A pharmacist compounded MDMA or lactose (placebo) into gelatin capsules to ensure all blinded capsules were similar in appearance and weight. The blind was broken for each participant after completion of all study assessments at the primary endpoint, which occurred approximately one month after the second blinded session.

### Procedures

At enrollment, before blinded sessions, participants prepared for experimental sessions with a male and female co-therapy team in three 60 to 90-min non-drug sessions. During these preparatory sessions, participants discussed feelings and life issues related to their diagnosis with an LTI, any questions or concerns associated with taking MDMA, including general hopes and fears, and any specific goals for their upcoming treatment. The therapists provided information about what to expect during blinded sessions. Participants completed several secondary outcome measures during preparatory sessions. There were four therapists organized into three co-therapy teams. Each participant saw the same co-therapy team for all visits.

After the third preparatory session, the first of two blinded experimental sessions occurred within five weeks of study enrollment. Experimental sessions occurred at two- to four-week intervals. Sessions were held in a comfortable, aesthetically pleasing living room, with an adjoining room where the participant would stay overnight. After pregnancy and drug screens were performed, each participant received either 125 mg of MDMA or the placebo during each experimental session, and an optional supplementary dose of 62.5 mg of MDMA or placebo was offered 1.5 to 2.5 h after the initial dose. The therapy team provided non-directive therapy throughout the 8-h sessions, as described in the treatment manual, which is detailed in “A Manual for MDMA-Assisted Psychotherapy in the Treatment of Anxiety Associated with a Life-Threatening Illness”^55^.

During each session, participants were provided with eyeshades and could listen to music through headphones to support reflecting on internal thoughts and emotions, as described in the Treatment Manual^55^. Participants were encouraged to “go within” their experience; to contemplate and engage with whatever they encountered during the session, trusting their “inner healing intelligence”. The therapists verbally checked in with participants at intervals during the session. If participants did not speak for an hour or appeared to be avoiding discussion of emotions or thoughts, the therapists reminded the participants about the session goals, and about addressing their LTI related anxiety and feelings. Physiological measures (blood pressure, heart rate, and temperature) were assessed every half hour for the first four hours, and hourly for the remainder of the experimental session. More frequent measurements were taken if any of these vital signs rose above pre-determined cut-off values. Participants remained at the study site overnight with a night attendant. At the end of each blinded session, the participant and each co-therapy team member indicated on a questionnaire their guess as to which condition they were assigned (placebo or MDMA) and degree of certainty on a 4-point Likert scale (1 = not at all certain and 4 = very certain), with participants and team members unaware of others’ guesses. Guesses were recorded to assess potential biases that might have been introduced due to perceived group assignments inducing a placebo effect. For example, a placebo participant who believes they might have received active MDMA might be more inclined to report positive effects, and vice versa, although blinding might also be less effective among active MDMA group participants due to the nature of the effects.

**Figure 3:**
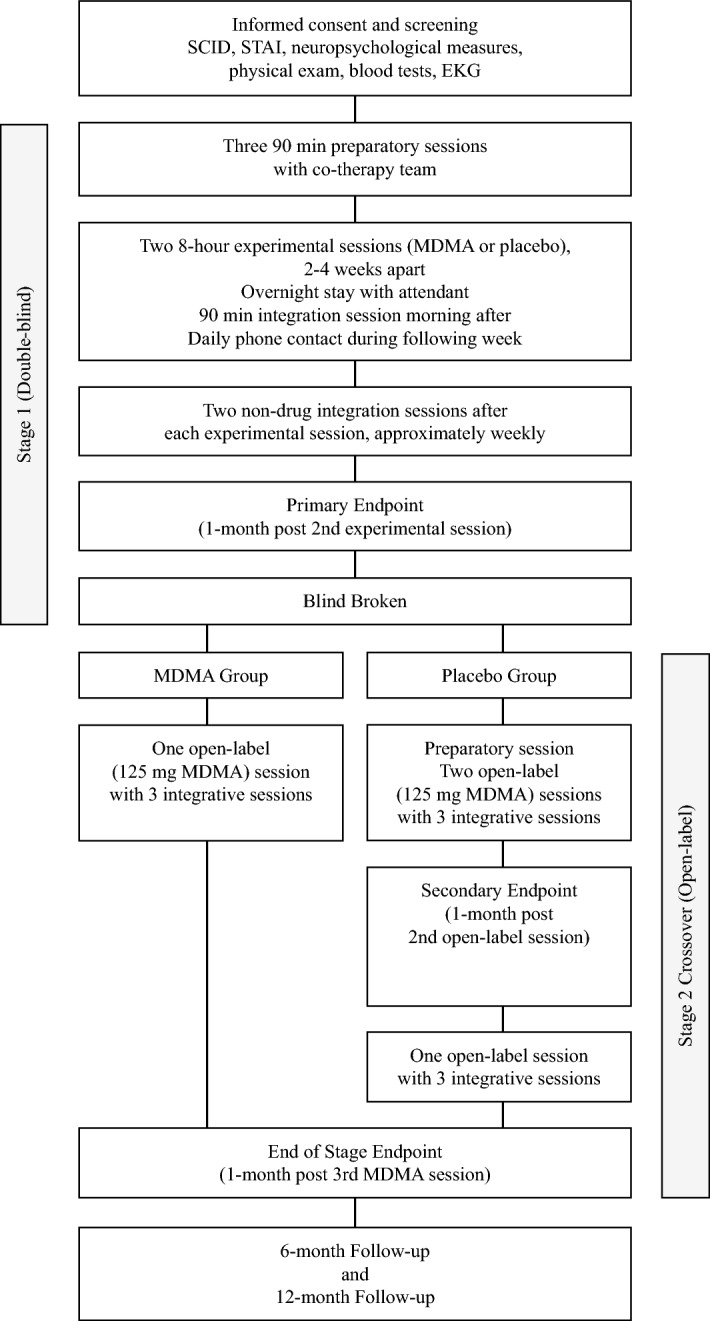
Study design.

The day following an experimental session, participants met with the therapy team for an integrative non-drug therapy session. One of the therapists contacted the participant by telephone for seven consecutive days after each experimental session to assess general well-being, and to record common, expected reactions and adverse events. Participants and the therapist team met for two integrative sessions during the month following, during which the participant continued to process material from experimental sessions. One month after the second experimental session, (primary endpoint), participants completed assessments of anxiety, depression, and subjective sleep quality, and an independent rater blind to group assignments assessed symptoms of depression with the Montgomery-Åsberg Depression Rating Scale (MADRS) and general psychological function via the Global Assessment of Functioning (GAF). The blind was broken at this point; participants randomized to MDMA received a third open-label MDMA psychotherapy session, and participants randomized to placebo crossed over into an open-label study arm (stage 2). Participants in the MDMA group were assessed a month after the third MDMA session (end of stage 1). The crossover consisted of three open-label sessions of 125 mg MDMA combined with associated non-drug psychotherapy sessions. Anxiety, depression and other symptoms were assessed one month after the second open-label MDMA experimental session (secondary endpoint) and one month after the third MDMA session (end of stage 2). See Fig. 1 CONSORT. All participants were re-assessed six and 12 months after their final experimental session with outcome measures administered at each follow-up visit (6-Month and 12-Month Follow-ups, Fig. 3).

### Outcomes

The STAI is a well-established and stable measure of cross-situational (trait) and current mood (state) anxiety^35^. The primary outcome measure of anxiety was change in the STAI-Trait subscale scores from baseline to primary endpoint. STAI-State anxiety scale total scores served as a secondary measure of anxiety. This self-reported measure is a 40-item questionnaire, which has been used in healthy populations as well as people with anxiety disorders.

Other secondary measures of anxiety, depression, and related symptoms included the Montgomery-Åsberg Depression Rating Scale (MADRS)^44^ (administered via an independent rater) and Beck Depression Inventory-II (BDI-II)^39,40^ for depression symptoms. Secondary measures also included self-reported sleep quality, assessed via Pittsburgh Sleep Quality Index (PSQI)^41^, perceived benefit or growth after a traumatic life event using the Post Traumatic Growth Inventory (PTGI)^36^, with the LTI serving as the reference event, a measure often used in populations with LTIs ^56^. Overall quality of life was assessed using Global Assessment of Functioning (GAF) (administered by independent rater) and the Functional Assessment of Chronic Illness Therapy Scale (FACIT-Sp)^42,43^, specifically designed for use in populations with LTIs. Mindfulness was measured with the Five Facet Mindfulness Questionnaire (FFMQ)^37,38^, attitudes concerning death with the Death Attitude Profile (DAP)^57^, and self-compassion was measured with the Self-Compassion Scale (SCS)^58^ [See eTable 6 for details and references].

Adverse events were collected throughout the study, except for events related to planned medical procedures or physician visits related to a medical diagnosis at baseline. Spontaneous reports of common expected events were collected during experimental sessions and for seven consecutive days following. The Columbia Suicide Severity Rating Scale (C-SSRS)^59^ was used to monitor suicidal ideation and behavior and administered during every in-person visit and on the second and seventh phone contact days.

### Statistical analyses

This was a feasibility study and therefore was not powered to detect statistical significance. The study design and sample size were based on Phase 2 studies of MDMA-assisted psychotherapy for PTSD treatment^18–20^; there was no prior MDMA research relevant to this population, or primary outcome measure to serve as a basis for power analysis. An intent-to-treat (ITT) analysis was conducted for safety and efficacy measures, which included all participants at a given time point. All analyses were set at an alpha level of 0.05 (two-tailed). The Folded F test was used to test equality of variances. Pooled *t* tests were reported for equal variances and Satterthwaite *t* tests for unequal variances.

The primary outcome analysis used an independent-samples *t* test to compare changes in STAI-Trait scores for measure of anxiety from baseline to one month after the second blinded experimental session (primary endpoint) across treatment groups. Analyses of secondary measures used the same approach. Hedges’ *g* independent-groups design was used to calculate effect sizes. Descriptive statistics were used to compare baseline characteristics and demographics.

Due to the small number of participants in the open-label crossover, treatment groups from each stage were combined since all participants had full dose MDMA in stage 1 or stage 2 for a pooled data set. Pooled data was analyzed using a one-way (time) repeated measures ANOVA. If main effects were found, within-subject Tukey’s pairwise tests were used to compare outcome scores at baseline, treatment exit after three MDMA sessions (MDMA group = end of stage 1; placebo group = end of stage 2), 6-month follow-up, and 12-month follow-up. Descriptive statistics of the open-label sessions are provided in the supplemental content including adverse events and outcome scores. Across the two blinded sessions, average peak vital signs (max recorded values during session post-drug) were compared between groups with *t* tests. Analyses were done using SAS, version 9.4 (SAS Institute, Cary, NC).

## Supplementary information

Supplementary Information.
