# Supplementary material for: MDMA-assisted psychotherapy for treatment of anxiety and other psychological distress related to life-threatening illnesses: a randomized pilot study
Source: Sci Rep. 2020 Nov 24;10:20442. doi: 10.1038/s41598-020-75706-1 (PMC7686344; doi:10.1038/s41598-020-75706-1)
Supplement: Supplementary file 1 — Supplementary Information. [file 41598_2020_75706_MOESM1_ESM.docx]

MDMA-Assisted Psychotherapy for Treatment of Anxiety and Other Psychological Distress Related to Life-threatening Illnesses: A Randomized Pilot Study

Philip E. Wolfson^1^, Julane Andries^1^, Allison A. Feduccia^2^, Lisa Jerome^2^, Julie B. Wang^2*^, Emily Williams^3^, Shannon C. Carlin^2^, Evan Sola^4^, Scott Hamilton^5^, Berra Yazar-Klosinski^6^, Amy Emerson^2^, Michael C. Mithoefer^7^, Rick Doblin^6^

^1^ Center for Transformational Psychotherapy, San Anselmo, CA, USA

^2^ MAPS Public Benefit Corporation, Santa Cruz, CA, USA

^3^ University of California, Department of Psychiatry, San Francisco, CA, USA

^4^ Private Practice, San Francisco, CA, USA

^5^ Stanford School of Medicine, Stanford Stroke Center, Palo Alto, CA, USA

^6^ Multidisciplinary Association for Psychedelic Studies, Santa Cruz, CA, USA

^7^ Medical University of South Carolina, Charleston, SC, USA

*** Correspondence:**Julie B. Wang, MPH, Ph.D.
[juliewang@mapsbcorp.com](mailto:alli@mapsbcorp.com)

Keywords: MDMA, psychedelics, MDMA-assisted psychotherapy, life-threatening illness, anxiety, depression, palliative care

**eTable 1. Frequency of Participants Reporting Treatment Emergent Adverse Events**

|  | **Blinded Placebo^a^**  **(n = 5)** | **Blinded MDMA^a^**  **(n = 13)** | **Open-label^b^**  **(n = 18)** | **Long-term Follow-up^c^**  **(n = 17)** |
| --- | --- | --- | --- | --- |
| Cardiac disorders | 0 | 0 | 1 (5.6) | 0 |
| Gastrointestinal disorders | 1 (20.0) | 4 (30.8) | 1 (5.6) | 0 |
| General disorders and administration site conditions | 1 (20.0) | 4 (30.8) | 0 | 0 |
| Infections and infestations | 1 (20.0) | 2 (15.4) | 2 (11.1) | 2 (11.1) |
| Injury, poisoning and procedural complications | 2 (40.0) | 3 (23.1) | 0 | 0 |
| Investigations | 0 | 1 (7.7) | 1 (5.6) | 0 |
| Musculoskeletal and connective tissue disorders | 1 (20.0) | 3 (23.1) | 0 | 2 (11.1) |
| Neoplasms benign, malignant and unspecified | 0 | 1 (7.7) | 0 | 2 (11.1) |
| Nervous system disorders | 1 (20.0) | 3 (23.1) | 1 (5.6) | 3 (16.7) |
| Psychiatric disorders | 1 (20.0) | 5 (38.5) | 5 (27.8) | 2 (11.1) |
| Renal and urinary disorders | 0 | 1 (7.7) | 0 | 0 |
| Reproductive system and breast disorders | 0 | 1 (7.7) | 0 | 0 |
| Respiratory, thoracic and mediastinal disorders | 0 | 1 (7.7) | 0 | 0 |
| Skin and subcutaneous tissue disorders | 0 | 2 (15.4) | 1 (5.6) | 0 |
| Vascular disorders | 0 | 0 | 1 (5.6) | 1 (5.6) |

^a^ Blinded treatment segment

^b^ Open-label sessions in Stage 1 or Stage 2; one participant stopped treatment after primary endpoint but AE collection continued

^c^ End of Stage 1 or End of Stage 2 to 12-month Follow-up; one participant stopped treatment after primary endpoint but AE collection continue

**eTable 2. Vital signs during Blinded Experimental Sessions**

| **Dose** |  | **Placebo**  **(n = 5)** | **MDMA**  **(n = 13)** | **Total**  **(n = 18)** | ***p*-value** |
| --- | --- | --- | --- | --- | --- |
| **Systolic Blood Pressure (mmHg)** | | | | | |
| Pre-drug  Mean (SD)  Min/Max |  | 127.5 (13.3)  102/145 | 129.9 (17.9)  91/178 | 129.3 (16.6)  91/178 |  |
| Peak  Mean (SD)  Min/Max |  | 146.0 (15.5)  126/173 | 157.5 (17.1)  127/192 | 154.3 (17.2)  126/192 | 0.07 |
| Final  Mean (SD)  Min/Max |  | 119.7 (8.7)  106/135 | 125.7 (11.4)  103/145 | 124.0 (11.0)  103/145 |  |
| **Diastolic Blood Pressure (mmHg)** | | | | | |
| Pre-drug  Mean (SD)  Min/Max |  | 78.5 (16.5)  46/94 | 78.7 (11.1)  55/106 | 78.6 (12.5)  46/106 |  |
| Peak  Mean (SD)  Min/Max |  | 91.6 (13.4)  72/112 | 93.9 (19.1)  (75/154) | 93.3 (17.6)  72/154 | 0.73 |
| Final  Mean (SD)  Min/Max |  | 78.4 (11.4)  64/100 | 73.1 (7.5)  58/84 | 74.6 (8.9)  58/100 |  |
| **Heart Rate (BPM)** | | | | | |
| Pre-drug  Mean (SD)  Min/Max |  | 69.0 (13.9)  50/100 | 69.3 (10.7)  53/95 | 69.2 (11.5)  50/100 |  |
| Peak  Mean (SD)  Min/Max |  | 81.5 (12.3)  71/108 | 105.5 (14.4)  89/133 | 98.9 (17.5)  71/133 | 0.14 |
| Final  Mean (SD)  Min/Max |  | 71.4 (7.1)  60/86 | 92.9 (16.4)  69/133 | 86.9 (17.4)  60/133 |  |
| **Body Temperature (°C)** | | | | | |
| Pre-drug  Mean (SD)  Min/Max |  | 36.3 (0.4)  35.7/36.9 | 36.3 (0.5)  35.6/37.4 | 36.4 (0.5)  35.6/37.4 |  |
| Peak  Mean (SD)  Min/Max |  | 36.9 (0.3)  36.5/37.5 | 37.3 (0.7)  36.1/39.9 | 37.2 (0.6)  36.10/39.9 | < 0.0001 |
| Final  Mean (SD)  Min/Max |  | 36.4 (0.6)  35.0/37.0 | 36.9 (0.4)  35.9/37.6 | 36.8 (0.5)  35.0/37.6 |  |

Abbreviations: Min, minimum; Max, maximum; mmHg, millimeters of mercury; °C, Celsius; BPM, beats per minute

**eTable 3. Suicidal Ideation and Behavior across the Treatment Period**

| **Experimental Session 1**  **No. (%)** | | | | | | | | |
| --- | --- | --- | --- | --- | --- | --- | --- | --- |
| **Dose** | | **Pre-**  **Drug^b^** | **During-**  **Drug**^c^ | **Integration Visit 1** | **Contact**  **Day 2** | **Contact Day 7** | **Integration**  **Visit 2** | **Integration**  **Visit 3** |
| Placebo | PI  SI  PB  N | 2 (40%)  0 (0)  1 (20%)  5 | 0 (0)  0 (0)  0 (0)  5 | 0 (0)  0 (0)  0 (0)  5 | 0 (0)  0 (0)  0 (0)  5 | 0 (0)  0 (0)  0 (0)  5 | 1 (20%)  0 (0)  0 (0)  5 | 1 (20%)  0 (0)  0 (0)  5 |
| MDMA | PI  SI  PB  N | 0  0 (0)  0 (0)  13 | 0 (0)  0 (0)  0 (0)  13 | 0 (0)  0 (0)  0 (0)  13 | 0 (0)  0 (0)  0 (0)  13 | 0 (0)  0 (0)  0 (0)  13 | 0 (0)  0 (0)  0 (0)  11 | 0 (0)  0 (0)  0 (0)  12 |
| **Experimental Session 2**  **No. (%)** | | | | | | | | |
| Placebo | PI  SI  PB  N | 0 (0)  0 (0)  0 (0)  5 | 0 (0)  0 (0)  0 (0)  5 | 0 (0)  0 (0)  0 (0)  5 | 0 (0)  0 (0)  0 (0)  5 | 0 (0)  0 (0)  0 (0)  5 | 0 (0)  0 (0)  0 (0)  5 | 0 (0)  0 (0)  0 (0)  5 |
| MDMA | PI  SI  PB  N | 0 (0)  0 (0)  0 (0)  13 | 0 (0)  0 (0)  0 (0)  13 | 0 (0)  0 (0)  0 (0)  13 | 0 (0)  0 (0)  0 (0)  13 | 0 (0)  0 (0)  0 (0)  13 | 0 (0)  0 (0)  0 (0)  13 | 1 (7.7%)  0 (0)  0 (0)  13 |
| **Experimental Session 3**  **No. (%)** | | | | | | | | |
| Open-label MDMA | PI  SI  PB  N | 0 (0)  0 (0)  0 (0)  11 | 0 (0)  0 (0)  0 (0)  10 | 0 (0)  0 (0)  0 (0)  12 | 0 (0)  0 (0)  0 (0)  12 | 0 (0)  0 (0)  0 (0)  12 | 0 (0)  0 (0)  0 (0)  12 | 1 (8.3%)  0 (0)  0 (0)  12 |
| **Experimental Session 4**  **No. (%)** | | | | | | | | |
| Placebo/  Open-label MDMA | PI  SI  PB  N | 0 (0)  0 (0)  0 (0)  5 | 0 (0)  0 (0)  0 (0)  5 | 0 (0)  0 (0)  0 (0)  5 | 0 (0)  0 (0)  0 (0)  5 | 0 (0)  0 (0)  0 (0)  5 | 0 (0)  0 (0)  0 (0)  5 | 0 (0)  0 (0)  0 (0)  5 |
| **Experimental Session 5**  **No. (%)** | | | | | | | | |
| Placebo/  Open-label MDMA | PI  SI  PB  N | 0 (0)  0 (0)  0 (0)  5 | 0 (0)  0 (0)  0 (0)  5 | 0 (0)  0 (0)  0 (0)  5 | 0 (0)  0 (0)  0 (0)  5 | 0 (0)  0 (0)  0 (0)  5 | 0 (0)  0 (0)  0 (0)  5 | 1 (20%)  0 (0)  0 (0)  5 |
| **Experimental Session 6**  **No. (%)** | | | | | | | | |
| Placebo/  Open-label MDMA | PI  SI  PB  N | 0 (0)  0 (0)  0 (0)  5 | 0 (0)  0 (0)  0 (0)  5 | 0 (0)  0 (0)  0 (0)  5 | 0 (0)  0 (0)  0 (0)  5 | 0 (0)  0 (0)  0 (0)  5 | 0 (0)  0 (0)  0 (0)  5 | 0 (0)  0 (0)  0 (0)  5 |

Abbreviations: C-SSRS, Columbia Suicide Severity Rating Scale; PI, Positive Ideation on C-SSRS; SI, Serious Ideation on C-SSRS; PB, Positive Behavior on C-SSRS; N, Number of Participants

^a^According to the C-SSRS scoring guide, scores of four or five on the suicidal ideation category are considered serious ideation, and scores of one or greater are considered positive behavior or ideation

^b^ Pre-drug measurement taken day of experimental session prior to drug administration

^c^ During-drug observation measured at experimental session endpoint, approximately 6 hours after drug administration

**eTable 4. Suicidal Ideation and Behavior at Endpoints^a^**

| **Study Endpoints** | | | | | | | | |
| --- | --- | --- | --- | --- | --- | --- | --- | --- |
| **Dose** | |  | **Primary**  **Endpoint** | **End of Stage 1** | **Secondary Endpoint** | **End of Stage 2** | **6-month Follow-up** | **12-month follow-up** |
| Placebo/  Open-label MDMA | PI  SI  PB  N |  | 0 (0)  0 (0)  0 (0)  5 | ---  ---  ---  --- | 0 (0)  0 (0)  0 (0)  5 | 0 (0)  0 (0)  0 (0)  5 | 0 (0)  0 (0)  0 (0)  5 | 1 (20%)  0 (0)  0 (0)  5 |
| MDMA | PI  SI  PB  N |  | 1 (7.7%)  0 (0)  0 (0)  13 | 0 (0)  0 (0)  0 (0)  12 | ---  ---  ---  --- | ---  ---  ---  --- | 0 (0)  0 (0)  0 (0)  12 | 0 (0)  0 (0)  0 (0)  12 |

Abbreviations: C-SSRS, Columbia Suicide Severity Rating Scale; PI, Positive Ideation on C-SSRS; SI, Serious Ideation on C-SSRS; PB, Positive Behavior on C-SSRS; N, Number of Participants

^a^According to the C-SSRS scoring guide, scores of four or five on the suicidal ideation category are considered serious ideation, and scores of one or greater are considered positive behavior or ideation

**eTable 5. Outcome Measures^a^ for Open-label sessions**

|  |  | **STAGE 1** | | | |  | **STAGE 2**  **(Open-label Crossover)** | | |
| --- | --- | --- | --- | --- | --- | --- | --- | --- | --- |
|  |  | **Primary Endpoint** | |  | **End of Stage 1** |  | **Secondary Endpoint** |  | **End of Stage 2** |
|  |  | Placebo  (n = 5) | MDMA  (n = 13) |  | MDMA  (n = 12) |  | Placebo/MDMA  (n = 5) |  | Placebo/MDMA  (n = 5) |
| ***Primary Efficacy Variable*** |  |  |  |  |  |  |  |  |  |
| STAI Trait Score, mean (SD) |  | 48.6 (12.6) | 38.9 (10.6) |  | 34.8 (8.6) |  | 44.4 (9.6) |  | 40.2 (11.3) |
| ***Secondary Efficacy Variables*** |  |  |  |  |  |  |  |  |  |
| STAI State Score, mean (SD) |  | 45.8 (12.5) | 37.5 (13.6) |  | 27.8 (6.5) |  | 36.4 (9.9) |  | 33.8 (10.2) |
| BDI-II, mean (SD) |  | 15.4 (9.9) | 9.3 (10.4) |  | 2.7 (1.8) |  | 6.0 (5.5) |  | 3.8 (4.0) |
| PSQI, mean (SD) |  | 6.8 (5.7) | 7.3 (4.5) |  | 5.7 (2.6) |  | 7.0 (6.0) |  | 6.2 (5.2) |
| PTGI, mean (SD) |  | 61.4 (24.9) | 71.0 (18.8) |  | 83.8 (13.1) |  | 73.6 (16.6) |  | 82.0 (10.7) |
| MADRS, mean (SD) |  | 12.2 (5.3) | 9.0 (9.0) |  | 4.1 (4.9) |  | 7.2 (5.6) |  | 4.2 (3.6) |
| GAF, mean (SD) |  | 72.8 (7.7) | 75.1 (9.9) |  | 81.7 (6.0) |  | 76.2 (8.1) |  | 82.8 (6.5) |
| SCS, mean (SD) |  | 2.7 (0.9) | 3.3 (0.6) |  | 3.6 (0.7) |  | 3.1 (0.8) |  | 3.5 (0.7) |
| FFMQ, mean (SD) |  | 3.3 (0.4) | 3.7 (0.5) |  | 3.9 (0.5) |  | 3.6 (0.5) |  | 3.7 (0.3) |
| DAP, mean (SD) |  |  |  |  |  |  |  |  |  |
| Fear of Death |  | 4.5 (0.7) | 3.7 (1.4) |  | 3.3 (1.6) |  | 4.6 (1.1) |  | 4.2 (1.3) |
| Death Avoidance |  | 2.4 (0.9) | 3.1 (1.6) |  | 2.8 (1.8) |  | 2.9 (1.1) |  | 2.3 (1.1) |
| Neutral Acceptance |  | 5.6 (0.5) | 5.9 (0.7) |  | 5.9 (0.6) |  | 5.8 (0.3) |  | 5.6 (0.6) |
| Approach Acceptance |  | 3.0 (0.7) | 3.5 (1.6) |  | 4.1 (1.4) |  | 3.2 (0.9) |  | 3.3 (0.7) |
| Escape Acceptance |  | 3.4 (1.3) | 3.9 (1.0) |  | 4.2 (0.9) |  | 4.1 (1.1) |  | 3.4 (0.7) |
| FACIT, mean (SD) |  |  |  |  |  |  |  |  |  |
| Physical Well-being |  | 21.4 (3.0) | 23.0 (4.3) |  | 25.2 (3.5) |  | 22.8 (4.9) |  | 24.6 (3.4) |
| Social and Family Well-being |  | 17.6 (6.3) | 18.5 (3.8) |  | 20.0 (3.8) |  | 18.4 (8.2) |  | 19.8 (6.1) |
| Emotional Well-being |  | 15.0 (3.9) | 16.3 (6.7) |  | 20.8 (2.0) |  | 17.0 (2.9) |  | 17.6 (3.4) |
| Functional Well-being |  | 18.8 (7.1) | 19.3 (6.3) |  | 21.8 (5.5) |  | 20.0 (6.1) |  | 21.8 (5.4) |
| Additional Concerns |  | 24.2 (10.3) | 28.5 (14.1) |  | 39.1 (7.6) |  | 27.0 (6.3)^b^ |  | 33.6 (8.9) |

Abbreviations: STAI, State-Trait Anxiety Inventory; BDI-II, Beck Depression Inventory-II; PSQI, Pittsburgh Sleep Quality Index; PTGI, Post Traumatic Growth Inventory; MADRS, Montgomery-Asberg Depression Rating Scale; GAF, Global Assessment of Functioning; SCS, Self-Compassion Scale; FFMQ, Five-Facet Mindfulness Questionnaire; DAP, Death Attitudes Profile; FACIT, Functional Assessment of Chronic Illness Therapy Scale

^a^ All outcomes are based on intent-to-treat set

^b^ Missing data (n=4)

**eTable 6. Assessments Used in Study**

| **Scale/Measure Name** | **Administration** | **Description** |
| --- | --- | --- |
| Structured Clinical Interview for DSM-IV-1-RV (SCID-IV) | Investigator administered and assessed | Structured diagnostic interview for assessing presence of psychiatric disorders, flexible and including items to assess included and excluded psychiatric disorders^1^. |
| State Trait Anxiety Inventory (STAI) | Self-report | The State Trait Anxiety Inventory (STAI) has two subscales ^2^, assessing “Trait” anxiety (trait anxiety, defined as long-standing nervous affect or anxiety disorder) and “State” anxiety (anxiety experienced in reaction to a specific environmental circumstance). Each subscale consists of 20 items, rated along a 4-point scale (1 = almost never/not at all to 4 = almost always/very much so), higher scores indicate greater anxiety. |
| Beck Depression Inventory II (BDI-II) | Self-report | The Beck Depression Inventory-II (BDI-II) is a 21-item self-report measure of depressive symptoms ^2^, with scores ranging from 0 to 63, higher scores indicative of greater (more severe) depression. |
| Global Assessment of Functioning (GAF) | Independent-rater assessed | The Global Assessment of Functioning (GAF) is a measure of global psychological function made via observation. Scores are on a 100-point measure, with higher scores indicative of greater psychological function. |
| Pittsburgh Sleep Quality Index (PSQI) | Self-report | The Pittsburgh Sleep Quality Index (PSQI) is 19-item self-report measure of sleep quality over a one-month period, with seven subscales (scales: sleep quality, sleep latency, sleep duration, habitual sleep efficiency, sleep disturbance, use of sleeping medications, and daytime dysfunction) and a total score ranging from 0-21, higher scores reflective of poorer sleep quality ^4^. |
| Montgomery-Asberg Depression Rating Scale (MADRS) | Independent-rater assessed | The Montgomery-Asberg Depression Rating Scale (MADRS) is a 10-item, clinician-administered questionnaire used to diagnose the severity of depressive episodes ^5^. Scores range from minimal (0-6) to severe (>34). |
| Post Traumatic Growth Inventory (PTGI) | Self-report | The Post Traumatic Growth Inventory (PTGI) is a 21-item self-report measure of perceived growth or benefits occurring after a traumatic event ^6^. It contains five subscales: relationship to others, new possibilities, personal strength, spiritual change, and appreciation of life. In this study, participants completed the PTGI in reference to the time since the trauma at baseline, and in reference to the beginning of their participation in the study on all subsequent occasions. |
| Functional Assessment of Chronic Illness Therapy Scale (FACIT-Sp) | Self-report | The Functional Assessment of Chronic Illness Therapy Scale (FACIT-Sp) is a 27-item self-report scale of quality of life issues specifically relevant to individuals with a chronic or life-threatening illness or condition; this version includes a 12-item Spirituality scale ^6,7^. |
| Death Attitudes Profile (DAP) | Self-report | The Death Attitudes Profile (DAP) is a 32-item self-report questionnaire that assesses  individual attitudes and beliefs about death and dying ^8,9^; items are marked on a 7-point scale from “strongly agree” to “strongly disagree.” The DAP divides items along 5 dimensions: fear of death, death avoidance, neutral acceptance, approach acceptance, and escape acceptance. |
| Five Facet Mindfulness Questionnaire (FFMQ) | Self-report | The Five-Facet Mindfulness Questionnaire (FFMQ) is a 39-item self-report questionnaire that assesses how often and to what degree respondents experience or utilize mindfulness in their everyday life ^10,11^. |
| Self-Compassion Scale (SCS) | Self-report | The Self-Compassion Questionnaire is a 26-item self-report questionnaire that assesses how respondents relate to themselves and treat themselves during difficult or painful experiences ^13,14^. Items are rated on a 5-point scale (1=almost never 5=almost always), and there are six scales: self-kindness, self-judgment, common humanity, isolation, mindfulness, and over-identification. |
| Long-Term Follow-up Questionnaire (LTFUQ) | Self-report | The Long-term Follow-up Questionnaire (LTFUQ) has been developed internally by the sponsor to assess long-term benefits and harms of MDMA-assisted psychotherapy at the six and 12- month follow-up visits. |
| Columbia Suicide Severity Rating Scale (C-SSRS) | Investigator-administered and rated | The Columbia Suicide Severity Rating Scale (C-SSRS) is a clinician-administered measure of suicidal ideation and behavior. It consists of a series of graded Yes/No questions about suicidal ideation, 5 items referring to when participant is feeling the most suicidal, responses on a 0-5 scale, and five Yes/No questions in reference to suicidal behavior ^14,15^. Scores include a 5-ponit score for suicidal ideation and a score for intensity of suicidal ideation, and a score for suicidal behavior, with higher scores indicating more severe or greater suicidal ideation or behavior. Suicidal ideation and behavior is summarized according to suggestions made in the Columbia-Suicide Severity Rating Scale Scoring and Data Analysis Guide ^17^. A positive response for suicidal ideation is counted when a subject answers “yes” to any one of the five suicidal ideation questions (Categories 1-5) on the C-SSRS, i.e. a score > 0 for suicidal ideation score. Serious suicidal ideation is a suicidal ideation score of 4 or 5. A positive response for suicidal behavior occurs when a subject answers “yes” to any one of the five suicidal behavior questions (Categories 6-10) on the C-SSRS, i.e. a score > 0 for suicidal behavior score. |
| Physiologic Vitals | Investigator-measured | During experimental sessions, blood pressure and heart rate were measured via automated sphigmometer every half-hour for the first four hours, then every hour until session end. Body temperature was measured at 60 minute intervals via tympanic thermometer. |
| Spontaneously Reported Reactions | Participant-reported, investigator recorded | Set of expected adverse events spontaneously reported by the participant during each experimental session and for seven days afterward. List based on reports in Phase 1 studies up through 2012, with several items added after observing reports from the initial study in people with PTSD. List included anxiety, diarrhea, difficulty concentrating, dizziness, drowsiness, dry mouth, fatigue, headache, heavy legs, impaired gait/balance, impaired judgment, increased irritability, insomnia, jaw clenching, lack of appetite, low mood, muscle tension, nausea, need more sleep, nystagmus, parasthesias, perspiration, restlessness, rumination, sensitivity to cold, thirst, weakness ^18-26^. |

**References**

1. First MB, Spitzer RL, Gibbon M, Williams JB. Structured clinical interview for DSM-IV-TR axis I disorders, research version, patient edition: SCID-I/P, 2002.

2. Spielberger CD, Gorsuch RL, Lushene RE, Vagg PR, Jacobs GA. Manual for the State-Trait Anxiety Inventory. Palo Alto, CA.: Consulting Psychologists Press; 1983.

3. Beck AT, Steer RA, Ball R, Ranieri W. Comparison of Beck Depression Inventories -IA and -II in psychiatric outpatients. *Journal of personality assessment* 1996; **67**(3): 588-97.

4. Buysse DJ, Reynolds CF, 3rd, Monk TH, Berman SR, Kupfer DJ. The Pittsburgh Sleep Quality Index: a new instrument for psychiatric practice and research. *Psychiatry research* 1989; **28**(2): 193-213.

5. Montgomery SA, Asberg M. A new depression scale designed to be sensitive to change. *Br J Psychiatry* 1979; **134**: 382-9.

6. Tedeschi RG, Calhoun LG. The Posttraumatic Growth Inventory: measuring the positive legacy of trauma. *Journal of traumatic stress* 1996; **9**(3): 455-71.

7. Cella D, Eton DT, Lai JS, Peterman AH, Merkel DE. Combining anchor and distribution-based methods to derive minimal clinically important differences on the Functional Assessment of Cancer Therapy (FACT) anemia and fatigue scales. *Journal of pain and symptom management* 2002; **24**(6): 547-61.

8. Cella D, Nowinski CJ. Measuring quality of life in chronic illness: the functional assessment of chronic illness therapy measurement system. *Archives of physical medicine and rehabilitation* 2002; **83**(12 Suppl 2): S10-7.

9. Gesser G, Wong PTP, Reker GT. Death attitudes across the life span: The development and validation of the Death Attitude Profile (DAP). *Omega (Westport)* 1987-1988; **18**: 113-28.

10. Reker GT, Peacock EJ, Wong PT. Meaning and purpose in life and well-being: a life-span perspective. *J Gerontol* 1987; **42**(1): 44-9.

11. Baer RA, Smith GT, Hopkins J, Krietemeyer J, Toney L. Using self-report assessment methods to explore facets of mindfulness. *Assessment* 2006; **13**(1): 27-45.

12. Baer RA, Smith GT, Lykins E, et al. Construct validity of the five facet mindfulness questionnaire in meditating and nonmeditating samples. *Assessment* 2008; **15**(3): 329-42.

13. Neff K. The Development and validation of a scale to measure self-compassion. *Self and Identity* 2003; **2**: 223-50.

14. Neff KD. The self-compassion scale is a valid and theoretically coherent measure of self-compassion. *Mindfulness* 2016; **7**(1): 264-74.

15. Posner K, Brown GK, Stanley B, et al. The Columbia-Suicide Severity Rating Scale: initial validity and internal consistency findings from three multisite studies with adolescents and adults. *The American journal of psychiatry* 2011; **168**(12): 1266-77.

16. Posner K, Oquendo MA, Gould M, Stanley B, Davies M. Columbia Classification Algorithm of Suicide Assessment (C-CASA): classification of suicidal events in the FDA's pediatric suicidal risk analysis of antidepressants. *The American journal of psychiatry* 2007; **164**(7): 1035-43.

17. Nilsson ME, Suryawanshi S, Gassmann C, Dubrava S, McSorley P, Jiang K. Columbia Suicide Severity Rating Scale Scoring and Data Analysis Guide. CSSRS Scoring Version 20. <http://www.cssrs.columbia.edu/documents/ScoringandDataAnalysisGuide_Feb2013.pdf>; 2013. p. 1-13.

18. Downing J. The psychological and physiological effects of MDMA on normal volunteers. *Journal of psychoactive drugs* 1986; **18**(4): 335-40.

19. Gamma A, Buck A, Berthold T, Liechti ME, Vollenweider FX. 3,4-Methylenedioxymethamphetamine (MDMA) modulates cortical and limbic brain activity as measured by [H(2)(15)O]-PET in healthy humans. *Neuropsychopharmacology* 2000; **23**(4): 388-95.

20. Greer G, Tolbert R. Subjective reports of the effects of MDMA in a clinical setting. *Journal of psychoactive drugs* 1986; **18**(4): 319-27.

21. Harris DS, Baggott M, Mendelson JH, Mendelson JE, Jones RT. Subjective and hormonal effects of 3,4-methylenedioxymethamphetamine (MDMA) in humans. *Psychopharmacology (Berl)* 2002; **162**(4): 396-405.

22. Hysek CM, Domes G, Liechti ME. MDMA enhances "mind reading" of positive emotions and impairs "mind reading" of negative emotions. *Psychopharmacology (Berl)* 2012; **222**(2): 293-302.

23. Hysek CM, Simmler LD, Ineichen M, et al. The norepinephrine transporter inhibitor reboxetine reduces stimulant effects of MDMA ("ecstasy") in humans. *Clinical pharmacology and therapeutics* 2011; **90**(2): 246-55.

24. Liechti ME, Saur MR, Gamma A, Hell D, Vollenweider FX. Psychological and physiological effects of MDMA ("Ecstasy") after pretreatment with the 5-HT(2) antagonist ketanserin in healthy humans. *Neuropsychopharmacology* 2000; **23**(4): 396-404.

25. Liechti ME, Gamma A, Vollenweider FX. Gender differences in the subjective effects of MDMA. *Psychopharmacology (Berl)* 2001; **154**(2): 161-8.

26. Vollenweider FX, Gamma A, Liechti M, Huber T. Psychological and cardiovascular effects and short-term sequelae of MDMA ("ecstasy") in MDMA-naive healthy volunteers. *Neuropsychopharmacology* 1998; **19**(4): 241-51.
